# Spondyloenchondrodysplasia Due to Mutations in *ACP5*: A Comprehensive Survey

**DOI:** 10.1007/s10875-016-0252-y

**Published:** 2016-03-08

**Authors:** Tracy A. Briggs, Gillian I. Rice, Navid Adib, Lesley Ades, Stephane Barete, Kannan Baskar, Veronique Baudouin, Ayse N. Cebeci, Philippe Clapuyt, David Coman, Lien De Somer, Yael Finezilber, Moshe Frydman, Ayla Guven, Sébastien Heritier, Daniela Karall, Muralidhar L. Kulkarni, Pierre Lebon, David Levitt, Martine Le Merrer, Agnes Linglart, John H. Livingston, Vincent Navarro, Ericka Okenfuss, Anne Puel, Nicole Revencu, Sabine Scholl-Bürgi, Marina Vivarelli, Carine Wouters, Brigitte Bader-Meunier, Yanick J. Crow

**Affiliations:** Manchester Centre for Genomic Medicine, Institute of Human Development, Faculty of Medical and Human Sciences, University of Manchester, Manchester, UK; St Mary’s Hospital, Central Manchester University Hospitals NHS Foundation Trust, Manchester Academic Health Science Centre, Manchester, UK; Department of Rheumatology, The Lady Cilento Children’s Hospital, Brisbane, Australia; Department of Clinical Genetics, The Children’s Hospital at Westmead, Sydney, Australia; Discipline of Paedatrics and Child Health, The University of Sydney, Sydney, Australia; Dermatology Department, Pitie-Salpetriere Hospital, Paris, France; Creighton University, 2500 California Plaza, NE 68178, Omaha, USA; Pediatric Nephrology Department, Robert Debré University Hospital – APHP, 48 boulevard Sérurier, 75019 Paris, France; Goztepe Educational and Research Hospital Pediatric Endocrinology Clinic, Istanbul, Türkiye; Pediatric Imaging Unit, Cliniques universitaires Saint-Luc, Université catholique de Louvain, Brussels, Belgium; Neuroscience Department, The Lady Cilento Children’s Hospital, Brisbane, Australia; School of Medicine, Griffith University, Gold Coast, Australia; Pediatric Rheumatology, Department of Pediatrics, University Hospitals Leuven, B-3000 Leuven, Belgium; Danek Gertner Institute of Human Genetics, Chaim Sheba Medical Center, Tel Aviv, Israel; Sackler School of Medicine, Tel Aviv University, Tel Aviv, Israel; Amasya University Medical Faculty, Department of Pediatric Endocrinology, Istanbul, Türkiye; Department of Pediatric Hematology and Oncology, Trousseau Hospital, Assistance Publique–Hôpitaux de Paris (APHP), Paris, France; Clinic for Pediatrics I, Inherited Metabolic Disorders, Medical University of Innsbruck, Anichstr. 35, A-6020 Innsbruck, Austria; J. J. M. Medical College, Davangere, Karnataka, 577004 India; Service de Virologie, AP-HP Hôpital Cochin, Paris, France; Department of Paediatrics, The Lady Cilento Children’s Hospital, Brisbane, Australia; Centre de Référence des Maladies Osseuses Constitutionnelles et Institut Imagine, Hopital Necker 149 rue de Sevres, 75015 Paris, France; APHP, Bicêtre Paris Sud, Department of Pediatric Endocrinology and Diabetology for Children, 94270 Le Kremlin Bicêtre, France; Reference Center for Rare Disorders of the Mineral Metabolism and Plateforme d’expertise Paris Sud Maladies Rares, APHP, 94270 Le Kremlin Bicêtre, France; Department of Paediatric Neurology, Leeds Teaching Hospitals NHS Trust, Leeds, UK; Epilepsy Unit, Pitie-Salpetriere Hospital, Paris, France; Kaiser Permanente – Genetics, 1650 Response Rd, Sacramento, CA 95815 USA; Génétique Humaine des Maladies Infectieuses, INSERM UMR 1163, Université Paris Descartes Sorbonne Paris Cité, Institut Imagine, Pièce 421-B1, 24 boulevard du Montparnasse, 75015 Paris, France; Centre for Human Genetics, Cliniques universitaires Saint-Luc, Université catholique de Louvain, Brussels, Belgium; Division of Nephrology, IRCCS Bambino Gesu’ Pediatric Hospital, Rome, Italy; Department of Microbiology and Immunology, Pediatric Immunology, KU Leuven, University of Leuven, Leuven, Belgium; Pediatric Immunology and Rheumatology Unit, Hôpital Necker, APHP, Paris, France; Institut Imagine, Paris, France; Laboratory of Neurogenetics and Neuroinflammation, Institut Imagine, 24 boulevard du Montparnasse, 75015 Paris, France

**Keywords:** Spondyloenchondrodysplasia, SPENCD/SPENCDI, *ACP5*, tartrate-resistant acid phosphatase (TRAP), type I interferon, interferon signature

## Abstract

**Purpose:**

Spondyloenchondrodysplasia is a rare immuno-osseous dysplasia caused by biallelic mutations in *ACP5.* We aimed to provide a survey of the skeletal, neurological and immune manifestations of this disease in a cohort of molecularly confirmed cases.

**Methods:**

We compiled clinical, genetic and serological data from a total of 26 patients from 18 pedigrees, all with biallelic *ACP5* mutations.

**Results:**

We observed a variability in skeletal, neurological and immune phenotypes, which was sometimes marked even between affected siblings. In total, 22 of 26 patients manifested autoimmune disease, most frequently autoimmune thrombocytopenia and systemic lupus erythematosus. Four patients were considered to demonstrate no clinical autoimmune disease, although two were positive for autoantibodies. In the majority of patients tested we detected upregulated expression of interferon-stimulated genes (ISGs), in keeping with the autoimmune phenotype and the likely immune-regulatory function of the deficient protein tartrate resistant acid phosphatase (TRAP). Two mutation positive patients did not demonstrate an upregulation of ISGs, including one patient with significant autoimmune disease controlled by immunosuppressive therapy.

**Conclusions:**

Our data expand the known phenotype of SPENCD. We propose that the OMIM differentiation between spondyloenchondrodysplasia and spondyloenchondrodysplasia with immune dysregulation is no longer appropriate, since the molecular evidence that we provide suggests that these phenotypes represent a continuum of the same disorder. In addition, the absence of an interferon signature following immunomodulatory treatments in a patient with significant autoimmune disease may indicate a therapeutic response important for the immune manifestations of spondyloenchondrodysplasia.

## Introduction

Spondyloenchondrodysplasia (SPENCD) (OMIM: 271,550) is a skeletal dysplasia, characterised by radiolucent metaphyseal and vertebral lesions [1]. Enchondromas predominantly develop within long bones, but may also occur in other areas of endochondral growth [2, 3]. The severity of the lesions varies, but slow progression during childhood is most frequently observed, resulting in significant short stature [2]. The first extra-osseous manifestations reported in SPENCD were of neurological dysfunction, including developmental delay and spasticity, which may be associated with intracranial calcification [2–5]. It was subsequently recognised that autoimmune disease was also a prominent feature [2, 3, 6].

SPENCD is caused by biallelic mutations in *ACP5*, encoding tartrate-resistant acid phosphatase (TRAP) [7, 8]. Affected individuals displayed an absence of TRAP serum expression and, in keeping with autoimmune manifestations, increased levels of serum interferon-alpha (IFNα) and an upregulation of interferon-stimulated genes (ISGs) (an interferon signature) [7]. An interferon signature is also recognised in SLE [9] and in monogenic disease in association with mutations in *TMEM173* [10] and *ISG15* [11] and any of the phenotypes recognised with mutations in *TREX1*, *RNASEH2A*/*B*/*C*, *SAMHD1*, *ADAR1* and *IFIH1* - including the monogenic encephalopathy Aicardi-Goutières syndrome (AGS), which can show significant overlap with SPENCD [12, 13].

Interestingly, since the description of causative *ACP5* mutations [7, 8] the disorder has been designated under two separate Online Mendelian Inheritance in Man (OMIM) entries, namely SPENCD (271,550) and SPENCD with immune dysregulation (SPENCDI) (607,944). SPENCD is described as a skeletal and neurological disorder of unknown aetiology. Whilst SPENCDI, refers to patients with an immune phenotype, in addition to the typical skeletal and neurological features, and is attributed to *ACP5* mutations.

Here, we present data from 26 patients with biallelic *ACP5* mutations conforming to both the SPENCD and SPENCDI phenotypes, leading us to propose that these phenotypes should be considered under the single designation of SPENCD.

## Methods

### Subjects

Twenty-six subjects with a clinical diagnosis of SPENCD (based on bone, brain and/or immune features, i.e. per current OMIM classification of SPENCD or SPENCDI) from 18 independent pedigrees were recruited through collaborating physicians. A U.K. Multicentre Research Ethics Committee (reference number 10/H1307/132) approved the study.

### Mutation Analysis

All coding exons of *ACP5* were sequenced as described previously [7]. Variant pathogenicity was analysed using Alumut and minor allele frequency was assessed using the National Heart, Lung, and Blood Institute (NHLBI) Exome Sequencing Project (ESP) database.

### Interferon Analysis

Type I interferon activity was determined using a cytopathic reduction assay [7]. As previously described [12], qPCR was performed on cDNA derived from whole blood and the median fold change of six interferon-simulated genes was compared with the median of the combined controls, to create an interferon score for each patient. Scores higher than the mean of the controls plus two SD (>2.466) were designated as a positive score.

## Results

### Mutation Data

**Fig. 1:**
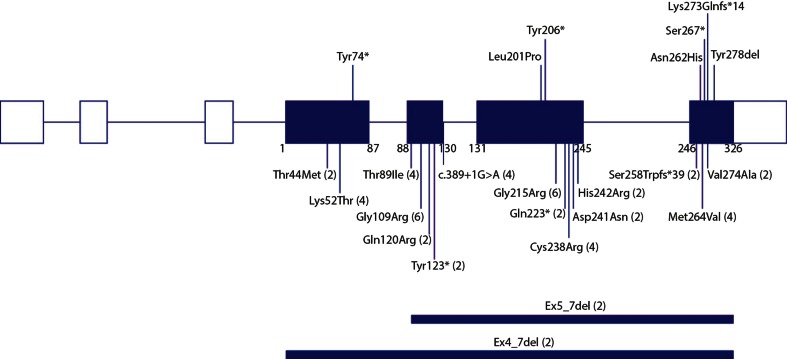
A diagram illustrating the distribution of all reported *ACP5* pathogenic variants. Below the gene diagram data are shown from this study with number of alleles per variant observed in parentheses; in addition pathogenic variants not identified in this study, but previously reported by Lausch et al. [8] and Girschick et al. [17] are depicted above the gene diagram

All 26 patients with a clinical diagnosis of SPENCD/SPENCDI harboured homozygous or compound heterozygous *ACP5* mutations (Table 1, Fig. 1). These data confirm the autosomal recessive nature of the disorder and suggest that it is not a genetically heterogeneous condition. The observation that 15 out of 18 families have a history of consanguinity is in keeping with the low minor allele frequency of pathogenic heterozygous variants in control populations. Seventeen different mutations distributed throughout the gene were identified (Fig. 1). Four mutations were observed in more than one pedigree, whilst the remainder were private to individual pedigrees.

**Table 1:** Demographic, genetic and presenting complaint of *ACP5* mutation positive patients

Patient^a^	Gender	Country of origin	Consanguinity	Relationship to other patients in cohort	Mutation	Protein	Age at presentation (months (mo.)/years (yr.))	Features at initial presentation	Current age in years
1	Female	France	No	None	11,544,822–11,556,767 del	p.Ex4_7 del Hom	3 yr.	Seizures	Deceased at age 30
2	Female	Austria	No	Sibling of 3	c.369C > A/c.721G > A	p.Tyr123X Het./p.Asp241Asn Het.	12 mo.	Delayed motor development	13
3	Male	Austria	No	Sibling of 2	c.369C > A/c.721G > A	p.Tyr123X Het./p.Asp241Asn Het.	3 yr.	AITP	13
4	Male	Turkey	Yes	Sibling of 5	c.266C > T	p.Thr89Ile Hom.	2 yr.	Spasticity & vasculitic skin rash	17
5	Female	Turkey	Yes	Sibling of 4	c.266C > T	p.Thr89Ile Hom.	14 yr.	Short stature	20
6	Male	Pakistan	Yes	None	c.667C > T	p.Gln223X Hom.	8 mo.	Short stature	17
7	Male	India	Yes	None	11,543,690–11,548,656 del	p.Ex5_7 del Hom	2 yr.	Recurrent infections	14
8	Female	Portugal	Yes	None^b^	c.791 T > A	p.Met264Val Hom.	3 yr.	Recurrent infections	34
9	Female	Mali	Yes	None	c.643G > A	p.Gly215Arg Hom.	6 yr.	Lupus nephropathy	22
10	Female	Egypt	Yes	None	c.772-790del	p.Ser258Trpfs*39 Hom.	4 yr.	Leg pain	17
11	Male	France	Yes	None	c.821 T > C	p.Val274Ala Hom.	2 yr.	Dev. delay & hypothyroidism	14
12	Female	Senegal	Yes	Sibling of 13	c.643 g > a	p.Gly215Arg Hom.	5 yrs.	Cerebral haemorrhage (AITP)	7
13	Male	Senegal	Yes	Sibling of 12	c.643 g > a	p.Gly215Arg Hom.	4 mo.	Metaphyseal lesions	2
14	Female	Turkey	Yes	None^c^	c.155 A > C	p.Lys52Thr Hom.	6 yrs.	Bruising & petechia (AITP)	12
15	Female	Mexico	Yes	None	c.725 A > G	p.His242Arg Hom.	3 yrs.	Short stature & leg bowing	16
16	Female	Turkey	Yes	Sibling of 17	c.155 A > C/c.790 A > G	p.Lys52Thr Hom./p.Met264Val Het.	2.5 yrs.	AIHA	15
17	Male	Turkey	Yes	Sibling of 16	c.155 A > C/c.790 A > G	p.Lys52Thr Hom./p.Met264Val Het.	3 mo.	Short stature & AIHA	13
18	Female	Iraq (Jewish)	Yes	None^d^	c.325G > A	p.Gly109Arg Hom.	2 yrs.	Short stature	36
19	Female	Lebanon	Yes	Sibling of 20	c.389 + 1G > A	p.? Hom.	9 yrs.	Jaundice (Hepatitis)	10
20	Female	Lebanon	Yes	Sibling of 19	c.389 + 1G > A	p.? Hom.	Birth	AITP	Deceased at less than 1 year
21	Male	Italy	Yes	None	c.359 A > G	Gln120Arg Hom.	1 mo.	AITP	9
22	Male	Israel (Arabic)	Yes	None^e^	c.325G > A	p.Gly109Arg Hom.	15 yrs.	Short stature & spasticity	35
23	Male	China	No	Sibling of 24, cousin of 25 & 26	c.325G > A/c.712 T > C	p.Gly109Arg Het./p.Cys238Arg Het.	5 yrs.	Short stature	12
24	Female	China	No	Sibling of 23, cousin of 25 & 26	c.325G > A/c.712 T > C	p.Gly109Arg Het./p.Cys238Arg Het.	7 mo.	Short stature & motor delay	7
25	Female	China	No	Sibling of 26, cousin of 23 & 24	c.131C > T/c.712 T > C	p.Thr44Met Het./p.Cys238Arg Het.	6 mo.	Short stature	10
26	Female	China	No	Sibling of 25, cousin of 23 & 24	c.131C > T/c.712 T > C	p.Thr44Met Het./p.Cys238Arg Het.	6 mo.	Short stature	15

*AITP* Autoimmune thrombocytopenia, *AIHA* Autoimmune haemolytic anaemia, *Dev.Delay* Developmental delay

^a^Patients 1 to 10 have been previously described [3, 4, 6, 7, 16] and additional data are added where available

^b^Clinically affected sibling previously described (Patient 2 [6])

^c^Affected cousin (confirmed *ACP5* biallelic mutation) previously described [8]

^d^Clinically affected sibling previously described (Patient 1 [4])

^e^Clinically affected sibling previously described (Patient 5 [4])

### Clinical Data

The most frequent reason for first seeking medical attention, in a total of 13 patients, was due to symptoms of immune disease, particularly autoimmune thrombocytopenia (AITP), which prompted presentation in five patients (Table 1). Skeletal manifestations, with short stature or leg pain/bowing, were the reason for initial presentation in 12 patients, whilst in six a neurological phenotype was manifest. In five individuals, complaints in more than one system prompted presentation. The age at which features first necessitated medical consultation varied from birth to 15 years.

The varied nature and severity of the disease continued throughout the disease course. For example, Patient 18 presented at two years of age with short stature. After which, she did not develop any additional features – so that at 36 years of age she was of normal intellect, in full time employment and gave birth to her first child. In contrast, Patient 1 presented at three years of age with seizures, and suffered a severe, progressive deterioration with skeletal, neurological and multi-organ autoimmune disease [3]. Marked variation was evident between the seven sib-pairs within the cohort, Patient 2 and 3 for example are non-identical twins and at 13 years of age Patient 2 was 5.5 SD below the mean for height, due to spasticity was classified as level 3 on the Gross Motor Classification System and manifested hypothyroidism and SLE with Class IV nephritis. In contrast Patient 3 was 2.5 SD below the mean for height, had no neurological manifestations and was diagnosed with eczema, AITP and proteinuria and did not fulfil lupus diagnostic criteria.

### Skeletal Manifestations

**Fig. 2:**
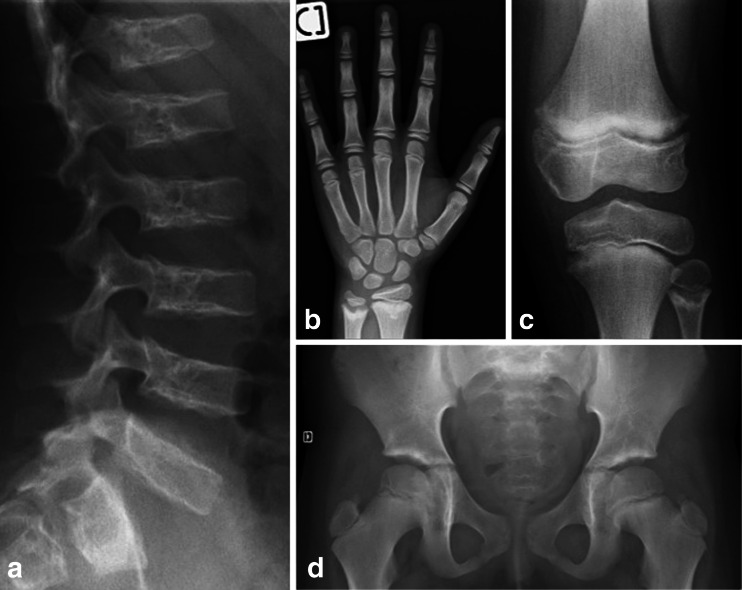
A skeletal survey in a patient with biallelic *ACP5* mutations. Imaging in Patient 14 at age seven years identified platyspondyly and lacunar lesions localized to the posterior third of the vertebral bodies (**a**); radiolucent lesions extending from the growth plate into the metaphysis of the long bones of the distal radius and ulna (**b**), proximal fibula and tibia (**c**) and the distal femora (**d**)

Until recently the diagnosis of SPENCD was made based on the presence of characteristic radiolucent metaphyseal and vertebral lesions (Fig. 2). Even with molecular testing, skeletal findings remain an important diagnostic clue. Thus, although short stature did not always lead to medical referral (perhaps because autoimmune or neurological features were more problematic), in all patients where assessment was undertaken, skeletal manifestations were evident on radiographs, with features of both platyspondyly and metaphyseal dysplasia in 23, and either platyspondyly or metaphyseal dysplasia in a further two (Table 2). We tested over 30 patients with a neurological and/or autoimmune phenotype, including AITP, SLE, intracranial calcification with seizures or spasticity, but without typical skeletal radiological changes, and did not identify biallelic *ACP5* mutations.

**Table 2:** Key skeletal and neurological features of *ACP5* mutation positive patients

Patient^1^	Last recorded height as SD below the mean (age at assessment)	Metaphyseal dysplasia	Platyspondyly	Intracranial calcification	Cranial MRI manifestations	Spasticity	Developmental delay (Degree)
1	4 (23)	Yes	Yes	Yes	Periventricular white matter changes	No	Yes (Mod.)
2	5.5 (12)	Yes	Yes	No	No	Yes	Yes (Mild)
3	2.5 (12)	No	Yes	NA	NA	No	No
4	4.3 (16)	Yes	Yes	Yes	No	Yes	No
5	3.8 (19)	Yes	Yes	Yes	Nil beyond changes associated with calcification	Yes	No
6	5 (13)	Yes	Yes	Yes	Ischaemic lateral ventricle lesions	Yes	Yes (Mod.)
7	3 (8)	Yes	Yes	Yes	Subcortical parietal granuloma (TB reaction)	No	No
8	4 (18)	Yes	Yes	NA	NA	No	No
9	6.5 (18)	Yes	No	Yes	No	No	No
10	3 (13)	Yes	Yes	NA	No	Yes	No
11	2 (12)	Yes	Yes	No	Ventricular dilatation	Yes	Yes (Severe)
12	2 (6)	Yes	Yes	Yes	Left subdural fronto-parietal subacute to chronic bleed; multiple petechial bilateral intra-parenchymal lesions	Yes	No
13	0 (0.5)	Yes	Yes	No	NA	No	No
14	2.5 (10)	Yes	Yes	NA	No	No	No
15	6 (12)	Yes	Yes	NA	NA	No	No
16	4 (12)	Yes	Yes	NA	NA	No	No
17	3 (10)	Yes	Yes	NA	No	No	No
18	3.5 (36)	Yes	Yes	No	NA	Yes	No
19	2.5 (10)	Yes	Yes	NA	Developmental venous anomaly associated with 3 mm pontine cavernoma	No	Yes (Mild)
20	NA	NA	NA	NA	NA	NA	NA
21	3 (10)	Yes	Yes	Yes	Leukodystrophy in the internal capsule of the semioval centre	Yes	Yes (Mild)
22	5.5 (35)	Yes	Yes	Yes	NA	Yes	Yes (Mod.)
23	2 (12)	Yes	Yes	NA	No	No	No
24	3 (7)	Yes	Yes	NA	No	Yes	No
25	3.5 (10)	Yes	Yes	NA	NA	No	No
26	5 (15)	Yes	Yes	NA	NA	No	No
Total	0–6.5	24/25 (1 NA)	24/25 (1 NA)	9/14 (12 NA)	8/16 (10 NA)	11/25 (1 NA)	7/25 (1 NA)
Further cases [8, 17]	1.8–4.2	15/15	14/15	6/8 (7 NA)	2/3 (13 NA)	4/14 (1 NA)	4/14 (1 NA)

*NA* Not assessed/reported, *Mod* moderate developmental delay

^1^Patients 1 to 10 have previously been described [3, 4, 6, 7, 16] and additional data are added where available

The skeletal phenotype is variable, with stature from within the normal range to 6.5 SD below the mean. Exacerbation of the degree of short stature over time was evident; for example, Patient 22 progressed from 3 to 5.5 SD below the mean between 15 and 35 years of age. Short stature was due to short trunk and/or short limbs depending upon the relative degree of spinal and/or long bone dysplasia. A number of patients also manifested specific skeletal deformities, including short distal phalanges (Patient 4 and 5), kyphosis and pectus carinatum (Patient 22) [4]. Bone mineral density was normal in Patient 2 and 19, whilst it was increased in Patient 4 and 5 (above superior percentile for age) and Patient 10 (1.5 SD above mean), although there was no evidence of localized osteopetrosis. Patient 17 demonstrated growth hormone (GH) deficiency and height improved with GH treatment. However, we are not aware of GH deficiency in other patients and indeed GH supplementation has been tried in at least one other patient without any effect on growth.

### Neurological Manifestations

Neurological features are frequent in *ACP5*-associated disease. We observed spasticity in eleven patients (44 %), and developmental delay in seven patients (28 %) (Table 2). Delay was typically of mild to moderate severity, except in Patient 11, who presented at age two years with hypothyroidism and severe delay. Additional neurological features included ataxia, seizures, psychosis and painful multifocal neuropathy.

**Fig. 3:**
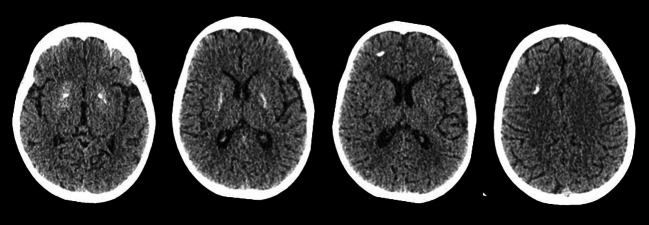
A cranial CT in a patient with biallelic *ACP5* mutations. Imaging in Patient 21 at nine years of age demonstrated intracranial calcification of the white and grey matter junction and bilateral blush and spots in the head of the caudate, putamen and globus pallidus and deep gyral matter on the right and left

Intracranial calcification was demonstrated on cranial CT imaging in nine out of the thirteen patients assessed (62 %), variably involving the basal ganglia, pons, dentate nuclei of the cerebellum and white-grey matter junction. Figure 3 demonstrates the intracerebral calcification observed in Patient 21, associated with a history of spasticity, developmental delay and autoimmune disease. Cranial MRI abnormalities were detected in 8 of 16 patients assessed with features including leukodystrophy (Table 2), and angiography in Patient 1 demonstrated an intracranial aneurysm.

Neurological manifestations may evolve over time. Patient 5 for example, was diagnosed at age 14 years due to short stature, Raynaud’s phenomenon and a positive family history [7]. At 19 years of age she developed lower limb pyramidal signs, which coincided with the detection of anti-nuclear antibodies for the first time, and a 10-fold increase in serum ISG levels compared to five years previously. CT revealed bilateral calcifications in the basal gangla and thalami.

### Immune Manifestations

Immune dysfunction was the most frequent presenting complaint in our cohort, and deterioration was observed over-time.

#### Autoimmune Manifestations

At least one autoimmune diagnosis was present in 22 patients (85 %), with many exhibiting more than three (Table 3). The most common was AITP in a total of 12 patients (46 %), seven of whom had SLE. AITP was lethal in one patient in infancy, and the associated morbidity in other cases was significant, with cerebral haemorrhage in two patients. In many cases, AITP necessitated intensive treatment with corticosteroids, intravenous immunoglobulins, rituximab and/or splenectomy. Seven patients with renal manifestations fulfilled ACR diagnostic criteria for SLE [14], whilst the other three were under follow up for proteinuria and did not fulfil criteria. In total nine (36 %) patients fulfilled SLE diagnostic criteria. Recurrent fevers of unknown origin were reported in several patients. Skin manifestations were noted, with severe eczema in four patients. Two patients had vasculitis; in Patient 1 sclerodermatous/acrocyanotic changes were evident in the hands and feet with oedema and sludging on capillaroscopy. Ultimately digital auto-amputation occurred, secondary to vasculitis. Skin biopsy in Patient 4 showed a perivascular polymorphonuclear infiltrate without evidence of deposition of complement or immunoglobulin, consistent with a non-specific leukocytoclastic vasculitis [3]. Two patients had Raynaud’s phenomenon and in one (Patient 5) capillaroscopy demonstrated the disappearance of parallel loops of some dilated capillaries with slow blood flow, which was not considered reminiscent of sclerodermatous disease [15]. Additional autoimmune phenotypes were reported, including in two patients: Sjögren’s syndrome and livedo reticularis; and in a single patient: post-operative macrophage activation syndrome, pancreatitis, scleroderma and polymyositis, coeliac disease, endocarditis and vitiligo. Disease progression was frequently observed; Patient 4, for example, developed renal lupus at age 15 years thus fulfilling the diagnostic criteria for SLE, and Patient 1 developed eight autoimmune diagnoses over a 27 year period.

**Table 3:** Key autoimmune features of *ACP5* mutation positive patients

Patient^1^	Antinuclear antibodies (fluorescence, titre)	Anti-dsDNA antibodies (titre)	Serum IFNα >2 IU/ml	ISG Score positive (>2.466)	Raynaud’s phenomenon (RP) or vasculitis	AITP (Anti-platelet abs)	AIHA	Juvenile idiopathic arthritis	SLE	Renal disease	Hypo- thyroidism	Biopsy proven autoimmune hepatitis
1	Yes (Nuclear dots & diffuse cytoplasmic, 1:1280)	No	Yes	Yes (40.034, 49.393)	Yes (vasculitis)	No	No	No	No	Yes	Yes	No
2	Yes (Homogenous,1:640)	Yes (1:320)	Yes	NA	No	No	No	No	Yes	Yes	Yes	No
3	Yes (Homogenous,1:5120)	Yes	Yes	Yes (13.931)	No	Yes	No	No	No	Yes	No	No
4	Yes (1:640)	Yes (100 Farr IU/ml)	Yes	Yes (14.826, 21.388)	Yes (vasculitis)	No	No	No	Yes	Yes	No	No
5	Yes (1:160)	No	Yes	Yes (3.756, 30.404)	Yes (RP)	No	No	No	No	No	No	No
6	Yes (>1:320)	Yes (1:1280)	Yes	Yes (71.094)	No	No	Yes	No	No	No	No	No
7	Yes (Strongly positive on immunoblot)	Yes (strongly positive on immunoblot)	NA	NA	No	Yes (Abs)	No	Yes	Yes	Yes	No	No
8	Yes (1:1280)	Yes (>500 Farr IU/ml)	NA	NA	No	Yes	No	No	Yes	No	Yes	No
9	Yes (1:1600)	Yes (121, *n* < 100 ELISA)	Yes	Yes (34.120)	No	Yes (Abs)	Yes	No	Yes	Yes	No	No
10	Yes (Speckled, 1:640)	Yes (33, *n* < 20 ELISA)	Yes	NA	No	No	No	No	No	Yes	No	No
11	Yes (Speckled, 1/200)	NA	NA	NA	No	Yes	No	No	No	No	Yes	No
12	Yes (1/200)	No	Yes	NA	No	Yes (Abs)	No	No	No	No	No	No
13	Yes (1/400)	Yes	Yes	NA	No	No	No	No	No	No	No	No
14	Yes (1:640)	Yes	NA	NA	No	Yes	No	No	No	No	Yes	No
15	Yes (Speckled, 1:80)	No	NA	NA	No	No	No	Yes (RF pos.)	No	No	No	Yes
16	No	No	NA	Yes (24.839)	No	No	Yes	No	No	No	No	No
17	Yes (Homogenous, 1/100)	Yes (1.78 index, *n* ≥ 1.1 positive)	NA	Yes (24.815)	Yes (RP)	No	Yes	No	Yes	Yes	No	No
18	NA	NA	NA	Yes (2.77)	No	No	No	No	No	No	No	No
19	Yes (Homogenous, 1/2560)	Yes (10 k IU/L)	NA	NA	No	No	No	No	No	No	No	Yes
20	NA	NA	NA	NA	NA	Yes	No	NA	NA	NA	NA	NA
21	Yes (Homogenous, 1:640)	Yes (1:640)	NA	No (0.725, 0.6)	No	Yes (No abs)	No	No	Yes	Yes	No	No
22	NA	NA	NA	No (0.770)	No	No	No	No	No	No	No	No
23	Yes (Homogenous, 1:640)	Yes (>90, *n* < 7 IU/ml)	NA	NA	No	Yes	Yes	No	Yes	Yes	No	LFTs abnormal, biopsy pending
24	NA	NA	NA	NA	No	Yes	No	No	No	No	No	LFTs abnormal, biopsy pending
25	Yes (Homogenous, >2560, *n* < 40)	Yes (15, *n* < 7 IU/ml)	NA	NA	No	Yes	Yes	No	Yes	No	No	Yes
26	Yes (Homogenous, 640, *n* < 40)	No	NA	NA	No	No	Yes	No	No	No	No	LFTs abnormal, biopsy pending
Total	21/22 (4 NA)	15/21 (5 NA)	10/10 (16 NA)	9/11 (15 NA)	4/25 (1NA)	12/26	7/26	2/25 (1 NA)	9/25 (1 NA)	10/25 (1 NA)	5/25 (1 NA)	3/25 (1 NA)
Further cases [8, 17]	7/14 (1 NA)	NA	5/5 10 NA	1/1 14 NA	1/14 1 NA	6/14 (1 NA)	3/14 (1 NA)	2/14 (1 NA)	5/14 (1 NA)	4/14 (1 NA)	1/14 (1 NA)	0/14 (1 LFT abnormal) (1 NA)

*NA* Not assessed/reported, *AITP* Autoimmune thrombocytopenia, *ABS* antibodies, *AIHA* Autoimmune haemolytic anaemia, *SLE* Systemic lupus erythematosus, *RF pos* Rheumatoid Factor Positive, *LFT* Liver Function Test

^1^Patients 1 to 10 have previously been described [3, 4, 6, 7, 16] and additional data are added where available

Serologically, 21 of 22 patients had at least one episode of positive antinuclear antibodies, and 15 of 21 positive anti-dsDNA antibodies. Positive antiplatelet, anti-phospholipid, antigliadin, antineutrophil, anti-thyroglobulin and anti ADAMTS XII antibodies were also detected. Two autoantibodies positive patients did not have clinical evidence of autoimmune disease.

**Fig. 4:**
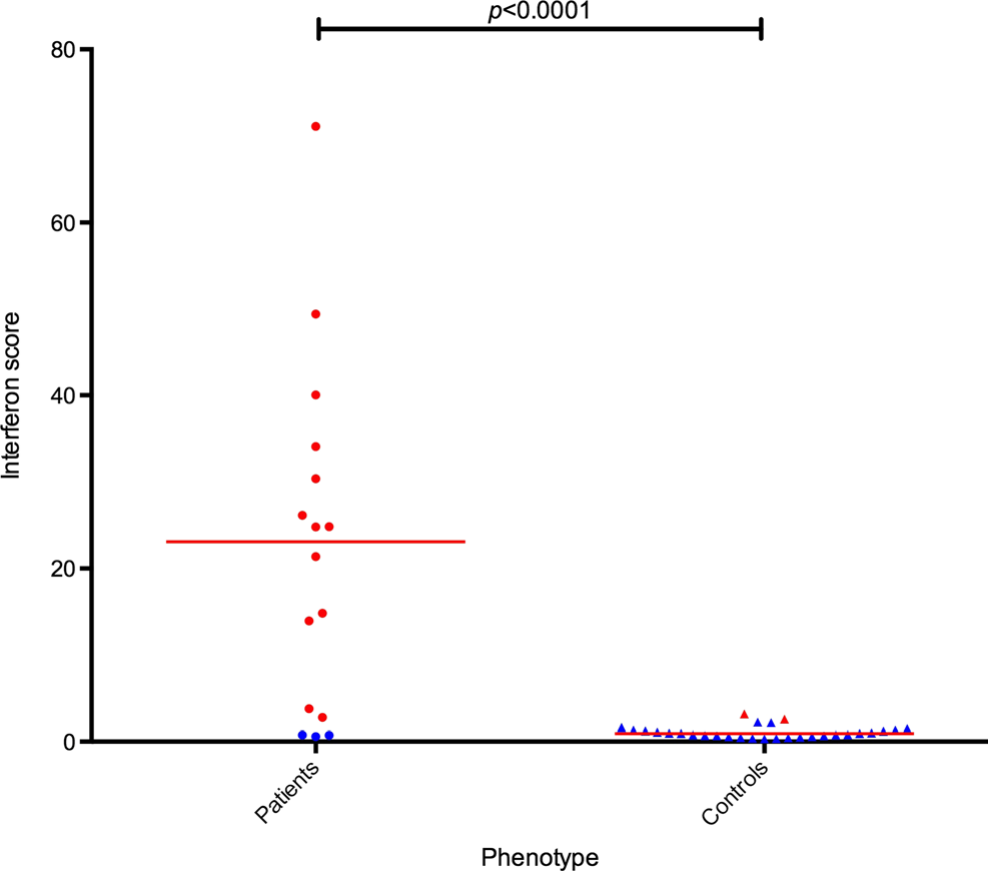
Expression of interferon-stimulated genes in SPENCD patients compared to controls. The median fold change for six ISGs (IFI27, RSAD2, IFI44L, ISG15, IFIT1, SIGLEC1) as normalised to 18S/HPRT1 is calculated to derive an interferon score. The mean interferon score of the controls plus two standard deviations above the mean (+ 2 SD) is considered as positive (red). Overall, the score in SPENCD patients was significantly higher than age and sex matched controls (** *p* < 0.0001)

We previously reported an association between biallelic *ACP5* mutations and elevated levels of serum IFNα and an upregulation of ISGs in whole blood [7]. In this cohort, all ten patients in whom serum IFNα activity levels were measured demonstrated elevated levels, and on serial assessment, levels were persistently high. An absence of CSF IFNα activity was observed in Patient 1, despite neurological manifestations [3]. ISGs were measured in 11 patients, and in nine the interferon score was significantly higher than age and sex matched controls and remained elevated on repeat measurement (Table 3 and Fig. 4). In Patient 5, the initial ISG assessment undertaken at age 14 years was marginally raised, however, a 10-fold increase was observed five years later, in association with neurological deterioration. Two patients, Patient 21 and Patient 22, did not demonstrate an interferon signature.

Patient 22 did not manifest any clinical autoimmune disease to age 35 years, when ISGs were within normal range (autoantibodies were not assessed). Features of SPENCD had been present since adolescence, with typical skeletal findings, spasticity, moderate intellectual disability and basal ganglia calcification [4].

Patient 21 had a history of skeletal, neurological and immune disease. He presented at one month of age with spontaneous bleeding and splenomegaly secondary to AITP. In the first year of life he suffered recurrent respiratory infections, but these resolved. He developed nephrotic syndrome at two years of age and, following the identification of class V lupus nephritis at four years of age, was treated with cyclosporine A for three years with partial remission. A subsequent relapse was managed with oral prednisone and cyclosporine A, but was then discontinued by the family. At nine years of age he developed right sigmoidal sinus thrombosis and severe hypertension, renal failure with class V lupus nephritis, serositis, (pleuritic and pericardial effusions), and dilated cardiomyopathy (ejection faction 34 %), with no sign of systemic infection. He had positive antinuclear and anti-dsDNA antibodies (1:640), low C3, C4 and thrombocytopenia. Two doses of intravenous methylprednisolone (2 mg/kg/day) and eight sessions of plasmapheresis were administered, followed by oral prednisone (2 mg/kg/day) for four weeks, which was then tapered (0.5 mg/kg/day) and given with mycophenolate mophetil (600 mg/m^2^/bd) and a dose of rituximab (375 mg/m^2^). Two months later he was switched to maintenance immunotherapy of prednisone (0.5 mg/kg/day) and mycophenolate mophetil (600 mg/m^2^/bd). This was continued for 12 months, and twice during this period (separated by several months) the ISG profile was assessed and an interferon signature was not detected. Follow-up during this time revealed significantly improved autoimmune disease with non-nephrotic proteinuria, low positive ANA titres and anti-dsDNA antibodies (1:80), with normal C3, C4 and white cell counts.

#### Manifestation of Immunodeficiency

Recurrent bacterial and viral infections were reported in five patients, raising the suggestion that immunodeficiency is a part of *ACP5*-associated disease (Table 4). Infections included recurrent pneumonia, disseminated herpes zoster, and skin and dental abscesses. Interpretation of immunological testing undertaken in the cohort was difficult, in terms of differentiating disease-related immunodeficiency from immune defects resulting from immunosuppressive therapy. Low lymphocyte count and/or hypogammaglobulinemia was evidenced in three patients who did not receive immunuppressive drugs and in nine treated patients (Table 4). Where lymphocyte subsets were available, the low counts consisted of low T, B and NK cell counts in two cases (one not treated and one treated), low T and B cell counts in one treated case, low B and NK cell counts in one treated case, low T and NK cell counts in one treated case and low T cell counts in five cases (two not treated and three treated). Of note, Patient 6 demonstrated a persistent hypogammaglobulinemia following rituximab treatment for AITP, whilst prior to treatment he had normal serum immunoglobulins.

**Table 4:** Features of immunodeficiency and total serum immunoglobulin and lymphocyte sub-sets in *ACP5* mutation positive patients

Patients^1^	History of recurrent infection	Medication at time of assay	IgG g/L	IgM g/L	IgA g/L	Lympho-cyte count	Neutrophil count	*CD3* (T-cells)	CD4+/CD3+	CD8+/CD3+	CD19 (total B cells)	CD56 & CD16 (NK cells)	CD3%	CD4+/CD3+ %	CD8+/CD3+ %	CD19%	CD56 & CD16%
1	No	Methotrexate	N	N	N	**1000** /mm3 (2500–7200)	**1000** /mm3 (1500–8000)	NA	NA	NA	NA	NA	NA	NA	NA	NA	NA
2	No	Mycophenol-ate mofetil & prednisolone	**5.1** (7.2–14.8)	**0.31** (0.7–2.8)	0.55 (0.5–2.6)	**3** g/L (4.5–13.5)	**1.6** g/L (2–7)	NA	NA	NA	NA	NA	NA	NA	NA	NA	NA
3	No	Prednisolone	NA	NA	NA	5.5 g/L (4.5–13.5)	2.5 g/L (2–7)	NA	NA	NA	NA	NA	NA	NA	NA	NA	NA
4	No	Azathioprine when Ig’s assayed & none for lymphocytes	19 (5.7–12.6)	1.1 (0.3–1.6)	4.56 (0.8–2.3)	4.04 X10(9)/L (1.5–7)	NA	3.10 X10(9)/L (0.66–2.41)	1.13 X10(9)/L (0.43–1.62)	1.605 X10(9)/L (0.15–1.01)	0.788 X10(9)/L (0.08–0.58)	0.376 X10(9)/L (0.05–0.52)	76.7 (52–78)	28 (25–48)	39.7 (9–35)	19.5 (8–24)	7.3 (4–17)
5	No	None	13.3 (5.7–12.6)	1.32 (0.3–1.6)	4.97 (0.8–2.3)	NA	NA	0.867 X10(9)/L (0.66–2.41)	0.518 X10(9)/L (0.43–1.62)	0.299 X10(9)/L (0.15–1.01)	0.245 X10(9)/L (0.08–0.58)	0.076 X10(9)/L (0.05–0.52)	72.2 (52–78)	43.2 (25–48)	24.9 (9–35)	20.4 (8–24)	6.3 (4–17)
6	No	Post-rituximab	**4.23** (6.5–12.2)	**0.48** (0.5–2.03)	2.38 (0.5–2.03)	3900/μl	NA	2769/μl (1200–1600)	1053/μl (650–1500)	1326/μl (370–1100)	780/μl (270–860)		71 (60–76)	27 (31–47)	34 (18–35)	20 (13–27)	
7	Yes (3 episodes of pneum-onia; dissemin-ated herpes zoster; TB reaction)	None	20.1 (3.4–12.3)	N	N	NA	NA	NA	**0.277** X10(9)/L (0.9–2.86)	**0.384** X10(9)/L (0.63–1.91)	NA	NA	NA	NA	NA	NA	NA
8	Yes (2 episodes of pneum-onia)	None	20.9 (6.7–17.3)	**0.05** (0.5–3.1)	1.5 (0.4–3.7)	2.7 X10(9)/L (1.5–7)	NA	NA	NA	NA	NA	NA	**32** (60–85)	**17** (30–60)	**13** (15–35)	NA	NA
9	No	Prednisolone, hydroxochlo-roquine & mycophenol-ate mofetil,	19 (6.4–13.5)	6.63 (0.7–3.1)	0.23 (0.6–3.5)	**736**/mm3 (2500–7200)	**1056**/mm3 (1500–8000)	**613**/mm3 (800–3500)	421/mm3 (400–2100)	**181**/mm3 (200–1200)	**26**/mm3 (200–600)	**66**/mm3 (70–1200)	NA	NA	NA	NA	NA
10	No	Prednisolone,	17.7 (6.5–12.2)	1.3 (0.5–2.0)	**0.23** (0.5–2.03)	NA	NA	NA	1080/μl (650–1550)	1800/μl (370–1100)	NA	N	NA	**27** (31–47)	45 (18–35)	NA	NA
11	Yes (severe chicken-pox; skin & dental abscesses)	None	13 (6.5–12.2)	0.36 (0.5–2.0)	1.56 (0.5–2.03)	1000/μl (2500–7200)	NA	670/μl (1200–2600)	200/μl (650–1550)	280/μl (370–1100)	**210**/μl (270–860)	60/μl (100–480)	67 (60–76)	**20** (31–47)	28 (18–35)	21 (13–27)	6 (4–17)
12	No	Steroids	23 (6.5–12.2)	1.9 (0.5–2.0)	1.1 (0.5–2.03)	**1000/**μl (2500–7200)	NA	NA	**300**/μl (700–2200)	**420**/μl (490–1300)	**80**/μl (390–1400)	NA	NA	NA	NA	NA	NA
13	No	None	NA	NA	NA	NA	NA	NA	NA	NA	NA	NA	NA	NA	NA	NA	NA
14	No	Prednisolone	13 (6.5–14.5)	1.56 (0.5–2)	2.09 (0.45–2.5)	3.9 X10(9)/L (0.8–5)	3.49 X10(9)/L (1.6–7)	NA	NA	NA	NA	NA	77	35	38	11	
15	No	Prednisolone & azathioprine	15.8 (6–16)	0.95 (0.3–1.9)	2.84 (0.4–3.75)	NA	NA	1084/μl (800–3500)	727/μl (400–2100)	333/μl (200–1200)	**131**/μl (200–600)	**13**/μl (70–1200)	89 (52–78)	60 (25–48)	27 (9–35)	11 (8–24)	**1** (6–27)
16	No	Prednisolone	38.8 (5.5–16.3)	0.6 (0.6–2.9)	1.75 (0.8–4.5)	2700/mm^3^ (2500–7200)	2200/mm^3^ (1500–8000)	NA	NA	NA	NA	NA	80.6 (57–86)	29.3 (29–57)	45.8 (13–47)	11.75 (3.5–15.5)	5.75 (4.5–30)
17	No	Prednisolone	NA	1.1 (0.6–2.9)	3.84 (0.8–4.5)	**1600**/mm^3^ (2500–7200)	3000/mm^3^ (1500–8000)	NA	NA	NA	NA	NA	63 (57–86)	**25.6** (29–57)	33.4 (13–47)	27.5 (3.5–15.5)	**4** (4.5–30)
19	Yes	Azathioprine	17 (6.2–14.4)	0.77 (0.5–2.6)	1.67 (0.7–2.9)	NA	NA	NA	NA	NA	NA	NA	NA	NA	NA	NA	NA
20	No	NA	NA	NA	NA	NA	NA	NA	NA	NA	NA	NA	NA	NA	NA	NA	NA
21	Yes (frequent RTI <1 yr.)	Prednisolone	NA	NA	NA	**1550**/mm^3^ (2500–7200)	7660//mm^3^ (1500–8000)	NA	NA	NA	NA	NA	**16.8** (57–86)	**4** (29–57)	**12** (13–47)	74.3 (3.5–15.5)	8.5 (4.5–30)
22	No	NA	NA	NA	NA	NA	NA	NA	NA	NA	NA	NA	NA	NA	NA	NA	NA
23	No	NA	NA	NA	NA	NA	NA	NA	NA	NA	NA	NA	NA	NA	NA	NA	NA
24	No	NA	NA	NA	NA	NA	NA	NA	NA	NA	NA	NA	NA	NA	NA	NA	NA
25	No	None	12.1 (5–13)	0.8 (0.5–1.6)	1.2 (0.4–1.7)	NA	NA	3.13 X10(9)/L (0.66–2.41)	1.23 X10(9)/L (0.43–1.62)	1.82 X10(9)/L (0.15–1.01)	0.59 X10(9)/L (0.08–0.58)	0.2 X10(9)/L (0.05–0.52)	79	31	46	15	5
26	No	NA	NA	NA	NA	NA	NA	NA	NA	NA	NA	NA	NA	NA	NA	NA	NA
Total	5/25 (1 NA)																
Further cases [8, 17]	1/15																

Bold and underlined values are below the normal reference range

^1^Immune features in Patients 7 and 8 have previously been described [6, 16]

*NA* Not assessed/reported, *N* Normal, *RTI* Respiratory tract infections

### Causes of Death and Severe Disability

Two patients died in our cohort, Patient 1 at age 30 years, following a gastrointestinal bleed and heart failure with severe arterial hypertension and Patient 20 before one year of age secondary to AITP. Disease morbidity was marked, with nine patients manifesting three or more different autoimmune diagnoses, which frequently required immunosuppressive therapy (Table 4), or in the case of AITP splenectomy. Mental retardation was typically of mild to moderate severity, but in Patient 11 this was categorized as severe. Whilst stature was variable, when 6.5 SD below the mean at 18 years this likely impeded activities of daily living.

## Discussion

This review describes the largest cohort of patients with confirmed biallelic *ACP5* mutations yet reported. Our findings highlight the marked clinical variability of the associated phenotype, ranging from infantile death secondary to AITP, to isolated skeletal dysplasia in an adult woman. Lausch et al. [8]. also noted variability, reporting both a 64 year old affected male and childhood mortality. The age at which features first became manifest ranged from birth to 15 years, similar to the ten month to 16-year range reported by previously [8]. Clinical diversity was observed both within and between families, and means that patients may present to different medical specialists, increasing possible diagnostic delay. Disease progression was observed frequently, indicating the need for continued follow up. As all *ACP5* mutations assessed to date appear functionally null, with an absence of TRAP expression in the blood [7], additional unidentified modifying genetic and/or environmental factors may play a role in the inter- and intra-familial variation of disease manifestations. At present, no specific genotype-phenotype correlation is evident.

Skeletal radiological findings were a consistent feature, thus serving as a key diagnostic handle. As was observed by Lausch et al. [8] who identified skeletal changes in all 14 patients they reported (Table 2). However, variability was observed, with subtle manifestations in some cases, and two patients demonstrating only metaphyseal or vertebral changes. A mild skeletal phenotype, with only ‘discrete metaphyseal changes’ at the wrist was also reported by Girschick et al. [17]. In keeping with this radiological diversity, a height within the normal range, particularly at a young age, does not exclude the diagnosis. We note an increased bone mineral density in three of five patients measured, which is perhaps analogous to the ostepetrosis observed in the *ACP5* knock-out mouse [18].

Over half of the cohort demonstrated features of neurological disease, the commonest being spasticity (44 %), with onset as early as age two years. It is noteworthy that SPENCD/SPENCDI demonstrates phenotypic overlap with the neuro-inflammatory interferonopathy AGS and patients with *ISG15* mutations, the hallmarks of which are intracranial calcification and neurological dysfunction [11, 12]. Furthermore, an elevated IFNα in both the serum and CSF has been reported in a nine year old with confirmed biallelic *ACP5* mutations [17]. Thus, whilst the etiology of the neurological disease is unclear, we consider it likely to be secondary to abnormal immune activation.

The prevalence of autoimmune disease in the cohort was high, with 92 % of patients demonstrating clinical or serological features of autoimmunity. The onset of autoimmune pathology was early, with cytopenia before a year of age in three patients, and with many patients developed three or more autoimmune diagnoses during childhood, demonstrating the need for careful monitoring. Cytopenia was the commonest manifestation (AITP in 46 %) and paediatric SLE was also frequent (36 %). We propose that in cases of severe autoimmune disease, particularly in a consanguineous family and/or in association with a short stature, SPENCD/SPENCDI should be considered as a potential diagnosis.

Whilst autoimmune disease was not observed in all cases of *ACP5* related disease, a diverse range of organ-specific and systemic autoimmune conditions were detected. Part of the explanation for these disorders likely relates to elevated type I interferon levels, which are associated autoimmune disease [19]. Elevated levels of serum IFNα and/or ISGs were recorded in the majority of patients tested (Table 3 and Fig. 4). We hypothesize that type I interferon is elevated in TRAP deficiency because TRAP may be a negative regulator of IFNα, perhaps via its action upon osteopontin. Osteopontin, a highly phosphorylated glycoprotein, forms a signalsome with TLR9 and MyD88 in mice and appears integral to IFNα production in murine plasmacytoid dendritic cells [20].

The absence of ISGs in Patient 21 during maintenance immunosuppressive therapy suggests that the treatment was effective at both a symptomatic and a biochemical level. The identification of an effective therapy, even if multimodal, is important, as SPENCD related autoimmune disease may be fatal. The normal ISG value during treatment also suggests that the assay might be used to evaluate targeted therapies.

The observation of an absence of an interferon signature in a patient without clinical autoimmune disease is of interest, and an assessment of similar cases (e.g. those in Table 5) is needed to determine whether this is a recurrent phenomenon. As we have observed elevation of ISG expression in association with disease progression (Patient 5), we recommend a low threshold for the investigation of immune symptoms in all SPENCD patients. In addition, whilst an ISG signature is not universal in SPENCD it remains a useful screening tool when considering the diagnosis, and may serve as a biomarker of disease progression and future treatments.

**Table 5:** Literature summary of cases of SPENCD in whom genetic testing has not been undertaken to our knowledge

Patient (Pt.)	Gender	Consanguinity (relationship to other patients)	SPENCD skeletal dysplasia	Short stature (standard Deviation (SD) below the mean)	Neurological disease	Intracranial calcification	Immune phenotype
Schorr et al. [1] Pt. 1	Male	Yes (sibling of Pt. 2 [1])	Yes	Yes	No	NR	NR
Schorr et al. [1] Pt. 2	Male	Yes (sibling of Pt. 1 [1])	Yes	Yes	No	NR	NR
Gustavson et al. [21] Pt. 1	Female	No (sibling of Pt. 2 [21])	Yes	Yes (−7.5SD at 16 years)	No	NR	NR
Gustavson et al. [21] Pt. 2	Male	No (sibling of Pt. 1 [21])	Yes	Yes (−6.5SD at 13 years)	No	NR	NR
Spranger et al. [22] Pt. 1	Male	No	Possibly^1^	Yes	No	NR	NR
Spranger et al. [22] Pt. 2	Male	NR	Possibly^2^	Yes	Dev. delay	NR	NR
Spranger et al. [22] Pt. 3	Male	No	Possibly^3^	Yes	Dev. delay	NR	NR
Sauvegrain et al. [23] Pt. 1	Female	No	Yes	Yes	No	NR	NR
Sauvegrain et al. [23] Pt. 5	Male	Yes	Yes	Yes	Dev. delay	NR	NR
Chagnon et al. [24]	Male	No	Yes	Yes	Spasticity	NR	NR
Azouz [25]	Male	No	Yes	Yes	No	NR	NR
Ziv et al. [31] Pt. 1	Male	No (sibling of Pt. 2 [31])	Yes	Yes	NR	NR	NR
Ziv et al. [31] Pt. 2	Female	No (sibling of Pt. 1 [31])	Yes	Yes	NR	NR	NR
Menger et al. [30] Pt. 1	Male	Yes (sibling of Pt. 2 [30])	Yes	Yes (−6SD at 12 years)	Dev. delay	NR	NR
Menger et al. [30] Pt. 2	Male	Yes (sibling of Pt. 1 [30])	Yes	Yes (−6SD at 6 years)	Dev. delay	NR	NR
Menger et al. [30] Pt. 3	Male	Yes (distant relative of Pt. 1 and 2 [30])	Yes	Yes (−1.5SD at 12 years)	No	NR	NR
Menger et al. [30] Pt. 4	Male	Yes	Yes	Yes (−2SD at 6 years)	Dev. delay & spasticity	NR	NR
Frydman et al. [4] Pt. 1	Male	Yes (sibling of Pt. 18 in present cohort)	Yes	Yes (−3.5SD at 8 years)	No	No	No
Frydman et al. [4] Pt. 3	Male	Yes	Yes	NR	NR	NR	NR
Frydman et al. [4] Pt. 5	Male	Yes (sibling of Pt. 22 in present cohort)	Yes	Yes (−4.4SD at 8 years)	Dev. delay & spasticity	Yes	No
Frydman et al. [4] Pt. 6	Male	No	Yes	Yes (−5.4SD at 8 years)	No	Yes	NR
Robinson et al. [28] Pt. 1	Male	No (grandson of Pt. 2 [28])	Yes	Yes (−3SD at 19 years)	No	NR	NR
Robinson et al. [28] Pt. 2	Male	NR (paternal grandfather of Pt. 1 [28])	Yes	Yes (−5.5SD at 86 years)	NR	NR	NR
Passwell et al. [32] Pt. 1	Male	Yes (sibling with skeletal phenotype, data limited)	Yes	Yes	NR	Yes	SLE
Passwell et al. [32] Pt. 2	Female	Yes (sibling with skeletal phenotype, data limited)	Yes	Yes	NR	NR	SLE
Passwell et al. [30] Pt. 3	Female	Yes	Yes	Yes	NR	NR	SLE
Uhlmann et al. [26] Pt. 1	Male	No	Yes	Yes (−4SD at 5 years)	No	NR	NR
Uhlmann et al. [26] Pt. 2	Male	No	Yes	Yes	NR	NR	NR
Tuysuz et al. [5] Pt. 1	Male	No (sibling of Pt. 2 [5])	Yes	Yes (−4.5SD at 9 years)	No	No	NR
Tuysuz et al. [5] Pt. 2	Female	No (sibling of Pt. 1 [5])	Yes	Yes (−1.5SD at 21 years)^4^	No	No	NR
Tuysuz et al. [5] Pt. 3	Male	NR	Yes	Yes (−4.5SD at 7 years)	Dev. delay	Yes	NR
Bhargava et al. [29] Pt. 1	Male	No (son of Pt. 2 [29])	Yes	Yes (−2.5SD at 13 years)	No	NR	NR
Bhargava et al. [29] Pt. 2	Female	No (mother of Pt. 1 [29])	Yes	Yes (−2.5SD at 42 years)	No	NR	NR

*Pt* Patient number from original publication, *Dev Delay* developmental delay, *NR* not recorded

^1^Enchondromatosis with irregular vertebral lesionss

^2^Enchondromatosis with irregular vertebral lesions

^3^Enchondromatosis with mild platyspondyly

^4^Two years of growth hormone therapy at 12 years

We also note a history of recurrent bacterial and/or viral infections in five patients, in some with associated reduced lymphocyte counts (Table 4). Additional cases of SPENCD with immunodeficiency may include some of the patients described by Roifman and Melamed [6], who manifested immunodeficiency, autoimmunity and spondylometaphyseal dysplasia. One patient in the Roifman and Melamed [6] series had a skeletal diagnosis of SPENCD on X-ray [2] and was found to have a homozygous *ACP5* mutation (Patient 8 described here). Patient 8 had a younger brother with metaphyseal sclerosis, a history of recurrent infections and AITP, he died of encephalitis at age three years [6]; molecular testing in this case and the other reported cases [6] has not been reported. The case reported by Girschick et al. [17] supports the association of concurrent autoimmunity and immunodeficiency with both autoantibodies and low CD4+, B and NK cell counts noted. The finding of recurrent infection in SPENCD is of interest given the susceptibility to *Staphylococcus aureus* infection noted in the *ACP5* knock-out mouse [27]. Whilst additional data are needed, we would recommend in the interim that patients with biallelic *ACP5* mutations should be monitored for an infectious susceptibility and should undergo lymphocyte phenotyping and serum immunoglobulin values prior to immunosuppressive therapy.

The identification of biallelic *ACP5* mutations in two patients without immune dysfunction is interesting; specifically, a p.Gly109Arg homozygous mutation in Patient 18 and 22. These unrelated patients were diagnosed in childhood (Patient 2 and 4 [4]), along with their similarly affected siblings (Patient 1 and 5 [4], Table 5). All four individuals were reviewed in their 30’s, and an absence of clinical autoimmune disease was observed. Mutation testing has not been undertaken in either sibling [4]. According to the OMIM classification, these patients satisfy SPENCD, rather than SPENCDI criteria. However, our data suggest that this separation is inappropriate, since we observed this same mutation, in the compound heterozygote state, in two individuals, both of whom had skeletal and autoimmune manifestations and one neurological sequelae (Patient 22 and 23), and this mutation was reported previously in four patients (two in the homozygous state) who demonstrated variable skeletal, neurological and autoimmune features [8]. Furthermore, Lausch et al., [8] described a 10 year old patient with a p.Gly215Arg homozygous *ACP5* mutation in whom the only extra-skeletal feature was of intracranial calcification, i.e. a patient with ‘SPENCD’, whilst, we and Lausch et al., [8] observed the same mutation in three unrelated patients, from two families, all of whom had autoimmune disease.

The suggestion that SPENCD and SPENCDI appear to be a continuum has implications for our understanding of the disease spectrum and thus patient management. A review of the literature since 1976 identified 33 cases (beyond those with *ACP5* mutations [2, 7, 8] or those reported by Roifman and Melamed [6]) with skeletal features consistent with a diagnosis of SPENCD. Nine of the 33 cases also manifested a neurological phenotype, and three were diagnosed with SLE (see Table 5). Of the 33 cases, from 25 families, there were seven sibling-pairs and ten consanguineous parental relationships, in keeping with autosomal recessive inheritance in at 29 cases. Biallelic *ACP5* mutations may be present in these 29 cases, although genetic testing is required and genetic heterogeneity cannot be excluded. Four further cases demonstrate possible autosomal dominant transmission [28, 29] and different genes may explain the aetiology in these cases. However, the possibility of a causative heterozygous *ACP5* mutation, due to haploinsufficiency, also requires consideration. Most parents heterozygous for an *ACP5* mutation in our cohort appear healthy; however, two parents had short stature, two had a history of neuropsychiatric illness, and one psoriasis. Before conclusions can be made, genetic testing is needed in these cases, and further assessment is required of heterozygote *ACP5* carriers.

Twelve of the 25 families reported in Table 5 are Jewish families of Iraqi origin [1, 4, 28, 30–32]. Patient 18 and Patient 22 are both from Israel and both harbor the same homozygous point mutation, and this same variant was previously reported in a case of Jewish ancestry [8]. This suggests both an increased prevalence of SPENCD in Israel, as noted previously [28], and raises the possibility of a common founder mutation.

## Conclusions

Biallelic *ACP5* mutations are primarily associated with skeletal, neurological and immune features, which can be highly variable in their manifestation and severity. Our data show that this diversity may include an absence of autoimmune disease, and indicate that SPENCD and SPENCDI are a continuum of the same condition. The majority of patients demonstrate elevated expression of ISGs, which therefore represents a useful diagnostic tool. SPENCD is a rare condition, but may be associated with significant childhood morbidity and mortality. Thus, there is a need for improved understanding of the underlying disease mechanism to facilitate the development of targeted therapies. In the interim, multimodal immunosuppression may be of benefit in certain cases.

### Authorship Contributions

TAB and GIR performed genetic and ISG analysis. TAB and YJC designed the project, TAB wrote the manuscript and YJC and BBM reviewed the manuscript. BBM provided clinical samples and data. PL performed the type I IFN cytopathic assays. JHL interpreted patient cranial imaging. The remaining authors provided clinical samples and data.

## References

[1] Schorr S, Legum C, Ochshorn M (1976). Spondyloenchondrodysplasia - enchondromatomosis with severe platyspondyly in 2 brothers. Radiology.

[2] Renella R, Schaefer E, LeMerrer M, Alanay Y, Kandemir N, Eich G, Costa T, Ballhausen D, Boltshauser E, Bonafe L, Giedion A, Unger S, Superti-Furga A (2006). Spondyloenchondrodysplasia with spasticity, cerebral calcifications, and immune dysregulation: clinical and radiographic delineation of a pleiotropic disorder. Am J Med Genet A..

[3] Navarro V, Scott C, Briggs TA, Barete S, Frances C, Lebon P, Maisonobe T, Rice GI, Wouters CH, Crow YJ (2008). Two further cases of spondyloenchondrodysplasia (SPENCD) with immune dysregulation. Am J Med Genet A..

[4] Frydman M, Barziv J, Premingershapiro R, Brezner A, Brand N, Benami T, Lachman RS, Gruber HE, Rimoin DL (1990). Possbile heterogeneity in spondyloenchondrodysplasia - quadriparesis, basal ganglia calcifications, and chondrocyte inclusions. Am J Med Genet.

[5] Tuysuz B, Arapoglu M, Ungur S (2004). Spondyloenchondrodysplasia: clinical variability in three cases. Am J Med Genet A.

[6] Roifman CM, Melamed I (2003). A novel syndrome of combined immunodeficiency, autoimmunity and spondylometaphyseal dysplasia. Clin Genet.

[7] Briggs TA, Rice GI, Daly S, Urquhart J, Gornall H, Bader-Meunier B, Baskar K, Baskar, S Baudouin V, Beresford MW, Black GC, Dearman RJ, de Zegher F, Foster ES, Frances C, Hayman AR, Hilton E, Job-Deslandre C, Kulkarni ML, Le Merrer M, Linglart A, Lovell SC, Maurer K, Musset L, Navarro V, Picard C, Puel A, Rieux-Laucat F, Roifman CM, Scholl-Burgi S, Smith N, Szynkiewicz M, Wiedeman A, Wouters C, Zeef LA, Casanova JL, Elkon KB, Janckila A, Lebon P, Crow YJ. Tartrate-resistant acid phosphatase deficiency causes a bone dysplasia with autoimmunity and a type I interferon expression signature. Nat Genet 2011;43:127–131. 10.1038/ng.748 PMC3030921 21217755

[8] Lausch E, Janecke A, Bros M, Trojandt S, Alanay Y, De Laet C, Hubner CA, Meinecke P, Nishimura G, Matsuo M, Hirano Y, Tenoutasse S, Kiss A, Rosa RF, Unger SL, Renella R, Bonafe L, Spranger J, Unger S, Zabel B, Superti-Furga A (2011). Genetic deficiency of tartrate-resistant acid phosphatase associated with skeletal dysplasia, cerebral calcifications and autoimmunity. Nat Genet.

[9] Baechler EC, Batliwalla FM, Karypis G, Gaffney PM, Ortmann WA, Espe KJ, Shark KB, Grande WJ, Hughes KM, Kapur V, Gregersen PK, Behrens TW (2003). Interferon-inducible gene expression signature in peripheral blood cells of patients with severe lupus. Proc Natl Acad Sci U S A.

[10] Liu Y, Jesus AA,, Marrero B, Yang D, Ramsey SE,, Montealegre Sanchez GA,, Tenbrock K, Wittkowski H, Jones OY, Kuehn HS,, Lee CC,, DiMattia MA,, Cowen EW,, Gonzalez B,, Palmer I,, DiGiovanna JJ,, Biancotto A,, Kim H,, Tsai WL,, Trier AM,, Huang Y,, Stone DL,, Hill S, Kim HJ,, St Hilaire C, Gurprasad S,, Plass N,, Chapelle D,, Horkayne-Szakaly I,, Foell D,, Barysenka A,, Candotti F,, Holland SM,, Hughes JD,, Mehmet H,, Issekutz AC,, Raffeld M,, McElwee J,, Fontana JR, Minniti CP,, Moir S,, Kastner DL,, Gadina M,, Steven AC,, Wingfield PT,, Brooks SR, Rosenzweig SD,, Fleisher TA,, Deng Z,, Boehm M,, Paller AS,, Goldbach-Mansky R (2014). Activated STING in a vascular and pulmonary syndrome. N Engl J Med.

[11] Zhang X, Bogunovic D, Payelle-Brogard B, Francois-Newton V, Speer SD, Yuan C, Volpi S, Li Z, Sanal O, Mansouri D, Tezcan I, Rice GI, Chen C, Mansouri N, Mahdaviani SA, Itan Y, Boisson, B, Okada S, Zeng L, Wang X, Jiang H, Liu W, Han T, Liu D, Ma T, Wang B, Liu M, Liu JY, Wang QK, Yalnizoglu D, Radoshevich L, Uzé G, Gros P, Rozenberg F, Zhang SY, Jouanguy E, Bustamante J, García-Sastre A, Abel L, Lebon P, Notarangelo LD, Crow YJ, Boisson-Dupuis S, Casanova JL, Pellegrini S. Human intracellular ISG15 prevents interferon-α/β over-amplification and auto-inflammation. Nature. 2015; 517:89–93. 10.1038/nature13801 PMC4303590 25307056

[12] Rice GI, Forte GM, Szynkiewicz M, Chase DS, Aeby A, Abdel-Hamid MS, Ackroyd S, Allcock R, Bailey KM, Balottin U, Barnerias C, Bernard G, Bodemer C, Botella MP, Cereda C, Chandler KE, Dabydeen L, Dale RC, De Laet C, De Goede CG, Del Toro M, Effat L, Enamorado NN, Fazzi E, Gener B, Haldre M, JP L, Livingston JH, Lourenco CM, Marques W, Oades PJ, Peterson P, Rasmussen M, Roubertie A, Schmidt JL, Shalev SA, Simon R, Spiegel R, Swoboda KJ,, Temtamy SA, Vassallo G, Vilain CN, Vogt J,, Wermenbol V, Whitehouse WP,, Soler D, Olivieri I, Orcesi S, Aglan MS, Zaki MS, Abdel-Salam GM, Vanderver A, Kisand K, Rozenberg F, Lebon P, Crow YJ (2013). Assessment of interferon-related biomarkers in Aicardi-Goutieres syndrome associated with mutations in TREX1, RNASH2A, RNASH2B, RNASH2C, SAMHD1 and ADAR: A case-control study. Lancet Neurol.

[13] Crow YJ, Chase DS, Lowenstein Schmidt J, Szynkiewicz M, Forte GM, Gornall HL, Oojageer A, Anderson B, Pizzino A, Helman G, Abdel-Hamid MS,, Abdel-Salam GM, Ackroyd S, Aeby A, Agosta G, Albin C, Allon-Shalev S, Arellano M, Ariaudo G, Aswani V, Babul-Hirji R, Baildam EM, Bahi-Buisson N, Bailey KM, Barnerias C, Barth M, Battini R, Beresford MW, Bernard G, Bianchi M, de Villemeur T B, Blair EM,, Bloom M,, Burlina AB, Carpanelli ML, Carvalho DR, Castro-Gago M, Cavallini A, Cereda C, Chandler KE, Chitayat DA, Collins AE, Sierra Corcoles C, Cordeiro NJ, Crichiutti G, Dabydeen L, Dale RC, D’Arrigo S, De Goede CG, De Laet C, De Waele LM, Denzler I, Desguerre I, Devriendt K, Di Rocco M, Fahey MC, Fazzi E, Ferrie CD, Figueiredo A, Gener B, Goizet C, Gowrinathan NR, Gowrishankar K, Hanrahan D, Isidor B, Kara B, Khan N, King MD, Kirk EP, Kumar R, Lagae L, Landrieu P, Lauffer H, Laugel V, La Piana R, Lim MJ, Lin JP, Linnankivi T, Mackay MT, Marom DR, Marques Lourenco C, McKee SA, Moroni I, Morton JE, Moutard ML, Murray K, Nabbout R, Nampoothiri S, Nunez-Enamorado N, Oades PJ, Olivieri I, Ostergaard JR, Perez-Duenas B, Prendiville JS, Ramesh V, Rasmussen M, Regal L, Ricci F, Rio M, Rodriguez D, Roubertie A, Salvatici E, Segers KA, Sinha GP, Soler D, Spiegel R, Stodberg TI, Straussberg R, Swoboda KJ, Suri M, Tacke U, Tan TY, te Water Naude J, Wee Teik K, Thomas MM, Till M, Tonduti D, Valente EM, Van Coster RN, van der Knaap MS, Vassallo G, Vijzelaar R, Vogt J, Wallace GB, Wassmer E, Webb HJ, Whitehouse WP, Whitney RN, Zaki MS, Zuberi SM, Livingston JH, Rozenberg F, Lebon P, Vanderver A, Orcesi S, Rice GI (2015). Characterization of human disease phenotypes associated with mutations in TREX1, RNASH2A, RNASH2B, RNASH2C, SAMHD1, ADAR and IFIH1. Am J Med Genet A.

[14] Tan EM, Cohen AS, Fries JF, Masi AT, McShane DJ, Rothfield NF, Schaller JG, Talal N, Winchester RJ (1982). Special article - the 1982 revised criteria for the classification of systemic lupus erythematosus. Arthritis Rheum.

[15] Dolezalova P, Young S, Bacon P, Southwood T (2003). Nailfold capillary microscopy in healthy children and in childhood rheumatic diseases: a prospective single blind observational study. Ann Rheum Dis.

[16] Kulkarni ML, Baskar K, Kulkarni PM (2007). A syndrome of immunodeficiency, autoimmunity, and spondylometaphyseal dysplasia. Am J Med Genet A..

[17] Girschick H, Wolf C, Morbach H, Hertzberg C, Lee-Kirsch MA (2015). Severe immune dysregulation with neurological impairment and minor bone changes in a child with spondyloenchondrodysplasia due to two novel mutations in the ACP5 gene. Pediatr Rheumatol.

[18] Hayman AR, Jones SJ, Boyde A, Foster D, Colledge WH, Carlton MB, Evans MJ, Cox TM (1996). Mice lacking tartrate-resistant acid phosphatase (ACP5) have disrupted endochondral ossification and mild osteopetrosis. Development.

[19] Theofilopoulos AN, Baccala R, Beutler B, Kono DH (2005). Type I interferons (alpha/beta) in immunity and autoimmunity. Annu Rev Immunol.

[20] Shinohara ML, Lu L, Bu J, Werneck MB, Kobayashi KS, Glimcher LH, Cantor H (2006). Osteopontin expression is essential for interferon-alpha production by plasmacytoid dendritic cells. Nat Immunol.

[21] Gustavson KH, Holmgren G, Probst F (1978). Spondylometaphyseal dysplasia in 2 sibs of normal parents. Pediat Radiol..

[22] Spranger J, Kemperdieck H, Bakowski H, Opitz JM (1978). 2 peculiar types of enchondromatosis. Pediatr Radiol.

[23] Sauvegrain J, Maroteaux P, Ribier J, Garel L, Tato L, Rochiccioli P, De Magalhaes J, Duhamel B (1980). Multiple chondroma affecting the spine: spondylo-enchondroplasia and other forms. J Radiol.

[24] Chagnon S, Lacert P, Blery M (1985). Spondylo-enchondrodysplasia. J Radiol.

[25] Azouz EM (1987). Case report - 418 - multiple enchondromatosis (ollier disease) with severe vertebral changes. Skelet Radiol.

[26] Uhlmann D, Rupprecht E, Keller E, Hormann D (1998). Spondyloenchondrodysplasia: several phenotypes - the same syndrome. Pediatr Radiol.

[27] Bune AJ, Hayman AR, Evans MJ, Cox TM (2001). Mice lacking tartrate-resistant acid phosphatase (ACP5) have disordered macrophage inflammatory responses and reduced clearance of the pathogen, *Staphylococcus aureus*. Immunology.

[28] Robinson D, Tieder M, Copeliovitch L, Halperin N (1991). Spondyloenchondrodysplasia - a rare cause of short-trunk syndrome. Acta Orthop Scand.

[29] Bhargava R, Leonard NJ, Chan AKJ, Spranger J (2005). Autosomal dominant inheritance of spondyloenchondrodysplasia. Am J Med Genet A..

[30] Menger H, Kruse K, Spranger J (1989). Spondyloenchondrodysplasia. J Med Genet.

[31] Ziv N, Grunebaum M, Kornreich L, Mimouni M (1989). Case-report 512. Skelet Radiol.

[32] Passwell JH, Robinson G, Lotan D. (1991) Spondyloepiphyseal dysplasia associated with systemic lupus erythematosus: A new syndrome. Progress in immunodeficiency III, edited by Chapel HM, Levinsky RJ, Webster ADB. Royal Society of Medicine Services International Congress and Symposium Series No. 173, Royal Society of Medicine Services Limited.

